# Inhibition of Cdk8/Cdk19 Activity Promotes Treg Cell Differentiation and Suppresses Autoimmune Diseases

**DOI:** 10.3389/fimmu.2019.01988

**Published:** 2019-08-20

**Authors:** Zengli Guo, Gang Wang, Yanfang Lv, Yisong Y. Wan, Junnian Zheng

**Affiliations:** ^1^Lineberger Comprehensive Cancer Center, University of North Carolina at Chapel Hill, Chapel Hill, NC, United States; ^2^Department of Microbiology and Immunology, University of North Carolina at Chapel Hill, Chapel Hill, NC, United States; ^3^Cancer Institute, Xuzhou Medical University, Xuzhou, China; ^4^Center of Clinical Oncology, Affiliated Hospital of Xuzhou Medical University, Xuzhou, China

**Keywords:** Treg cells, Cdk8/Cdk19, Foxp3, TGF-β signaling, experimental autoimmune encephalomyelitis (EAE)

## Abstract

Foxp3 expressing regulatory T (Treg) cells, as the central negative regulator of adaptive immune system, are essential to suppress immune response and maintain immune homeostasis. However, the function of Treg cells is frequently compromised in autoimmunity and hyper-activated in infections and tumor microenvironments. Thus, manipulating Treg cells becomes a promising therapeutic strategy for treating various diseases. Here we reported that inhibition of Cdk8/Cdk19 activity by small molecule inhibitors CCT251921 or Senexin A greatly promoted the differentiation of Treg cells and the expression of Treg signature genes, such as Foxp3, CTLA4, PD-1, and GITR. Mechanistically, we found that the augmented Treg cell differentiation was due to sensitized TGF-β signaling by Cdk8/Cdk19 inhibition, which was associated with attenuation of IFN-γ-Stat1 signaling and enhancement of phosphorylated Smad2/3. Importantly, treatment with Cdk8/Cdk19 inhibitor CCT251921 significantly increased Treg population and ameliorated autoimmune symptoms in an experimental autoimmune encephalomyelitis (EAE) model. Taken together, our study reveals a novel role of Cdk8/Cdk19 in Treg cell differentiation and provides a potential target for Treg cell based therapeutics.

## Introduction

Treg cells are the central negative regulator of adaptive immune system, which is essential for immune tolerance and immune homeostasis (1). However, the function of Treg cells is frequently compromised in autoimmunity and hyper-activated in tumor microenvironments. Targeting Treg cells becomes a promising therapeutic strategy to treat many diseases (2). By upregulating Treg cell function, it can be used to treat autoimmunity such as multiple sclerosis, graft-vs.-host disease, systemic lupus erythematosus (SLE), and rheumatoid arthritis (RA). Down-regulation of Treg cells can be applied to boost immune system to fight against infection and cancer. Thus, understanding the molecular regulation of Treg differentiation will provide valuable targets for modulating Treg cell function.

As the master transcriptional factor of Treg cells, the induction and maintenance of Foxp3 expression is essential for Treg function to maintain immune homeostasis (3). Extensive studies have revealed that various transcriptional factors and pathways were involved in regulating Foxp3 expression. Among those, the transforming growth factor-β (TGF-β) induced Smad2/Samd3 phosphorylation is critical for the transcription of Foxp3 (4). Although, it is well known that TGF-β signaling is indispensable for *in vitro* Treg cell differentiation and function, how TGF-β signaling is regulated and modified by other signaling pathway is not fully understood.

Cdk8 and its paralog Cdk19 were identified as two kinases associated with the Mediator complex. As a general transcriptional factor, the Mediator complex regulates RNA polymerase II (RNAP II) activity (5). The Cdk8 module consists of Cdk8, cyclin C, MED12, and MED13. The kinase activity of Cdk8 is essential for its biological function. CDK8 has been identified as an oncogene in colon cancer by regulating β-catenin activity via its kinase activity (6). Also, CDK8 phosphorylates STAT1 at Ser727 in response to IFN-γ (7). Cdk8 has been reported to attenuate BMP signaling and TGF-β signaling by binding and phosphorylating the linker region of regulatory Smad proteins in tumor cells (8). As TGF-β signaling plays an essential role in the regulation of immune system (4), it will be necessary to explore whether and how Cdk8 regulates TGF-β signaling in T cells.

Recently, a number of potent inhibitors targeting the kinase activity of Cdk8 and Cdk19 have been developed, such as Cortistatin A (9), Senexin A (10), CCT251921 (11), MSC2530818 (12), and BRD6989 (13). Using small molecule inhibitor BRD6989, Johannessen et al. showed Cdk8/Cdk19 inhibition up-regulates IL-10 production by enhancing AP-1 activity in dendritic cells and macrophages (13), indicating that Cdk8/Cdk19 plays a role in regulating innate immunity. However, whether Cdk8/Cdk19 is involved in adaptive immune regulation has not been studied.

In this study, we found that inhibition of Cdk8/Cdk19 by small molecule inhibitors CCT251921 or Senexin A promoted Treg cell differentiation and the Treg signature gene expression, such as Foxp3, CTLA4, PD-1, and GITR. We demonstrated that the enhanced Treg differentiation was caused by sensitized TGF-β signaling with Cdk8/Cdk19 inhibition, which is partially dependent on the attenuated IFN-γ-Stat1 signaling and elevated Smad2 phosphorylation. In addition, *in vivo* study confirmed that treatment with Cdk8/Cdk19 inhibitor CCT251921 increased Treg population and mitigated autoimmune symptoms in EAE model. Taken together, our study for the first time reveals that Cdk8/Cdk19 is involved in adaptive immune regulation, especially in Treg cell differentiation, and could be used as a potential target for Treg cell based therapeutic strategy.

## Results

### Inhibition of Cdk8/Cdk19 Activity Promotes Treg Cell Differentiation

**Figure 1:**
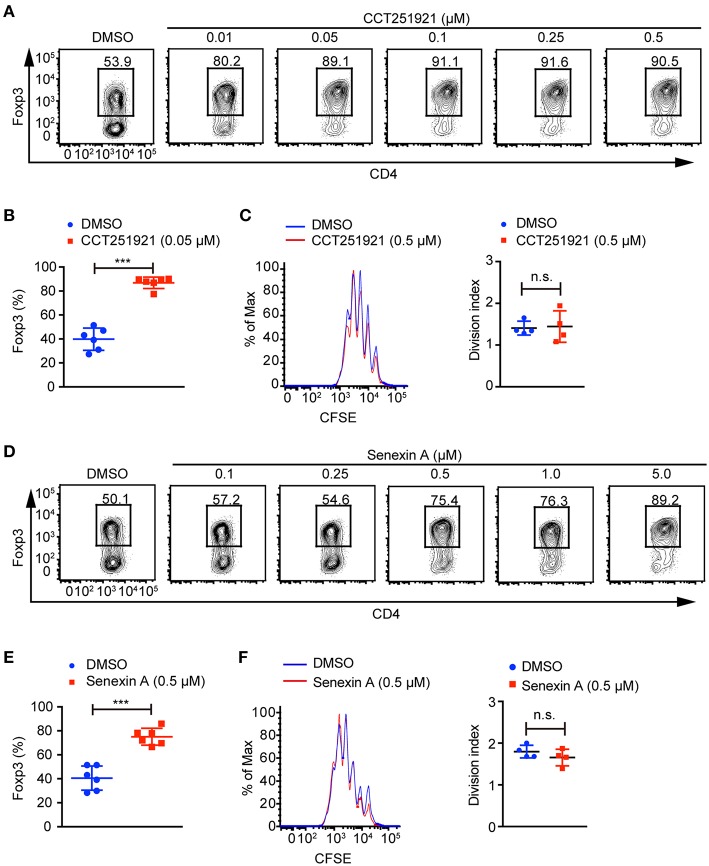
Cdk8/Cdk19 inhibition promotes the differentiation of Treg cells. **(A)** Purified CD4^+^ T cells were cultured under iTreg cell polarizing condition (1 ng/ml of TGF-β and 20 U/ml of rIL-2) in the presence of DMSO or different concentrations of CCT251921 as indicated. Treg population was assessed by Foxp3 intracellular staining and flow cytometry. The FACS plots are representative for three independent experiments. **(B)** Quantification of Foxp3^+^ cell percentage after DMSO or 0.05 μM CCT251921 treatment. Composite data of 7 mice per group from three independent experiments, Mean ± SD, ***P < 0.001, by two-sided *t*-test. **(C)** CFSE-labeled CD4^+^ T cells were activated with the treatment of mock (blue line) or CCT251921 (0.5 μM, red line) for 4 days. Cell proliferation was analyzed by CFSE dilution and division index (right) by flow cytometry. Result is summary of 4 mice per group from two independent experiments, Mean ± SD, n.s. not significant, by two-sided *t*-test. **(D)** Purified CD4^+^ T cells were cultured under iTreg cell polarizing condition in the presence of DMSO or different doses of Senexin A as indicated. Treg population was assessed by Foxp3 intracellular staining and flow cytometry. The FACS plots are representative for three independent experiments. **(E)** Quantification of Foxp3^+^ cell percentage after DMSO or 0.5 μM Senexin A treatment. Composite data of 6 mice per group from three independent experiments, Mean ± SD, ***P < 0.001, by two-sided *t*-test. **(F)** CFSE-labeled CD4^+^ T cells were activated with the treatment of mock (blue line) or Senexin A (0.5 μM, red line) for 4 days. Cell proliferation was analyzed by CFSE dilution and division index (right) by flow cytometry. Result is summary of 4 mice per group from two independent experiments, Mean ± SD, n.s. not significant, by two-sided *t*-test.

To investigate the role of Cdk8/Cdk19 in adaptive immunity, a potent and selective inhibitor CCT251921 (11) was used to block Cdk8/Cdk19 activity in the *ex vivo* T cell differentiation system. We found that CCT251921 treatment significantly increased Foxp3^+^ Treg cell population with a very low concentration of 10 nM and the beneficial effect saturated at 50 nM (Figures 1A,B). As Cdk8/Cdk19 inhibition has been reported to suppress cell proliferation in leukemia (14), we further analyzed the cell proliferation by CFSE labeling in the presence of CCT251921. The results showed that CCT251921 does not affect cell proliferation even at 500 nM in our experiment (Figure 1C). But the proliferation of T cells was dramatically inhibited when the dose of CCT251921 increased to 10 μM or higher (data not shown). To further confirm the promotion of Foxp3^+^ Treg cell differentiation is dependent on Cdk8/Cdk19 inhibition, another selective and structurally different Cdk8/Cdk19 inhibitor Senexin A (10) was used in the *ex vivo* T cell differentiation system. As expected, treatment with Senexin A also promoted Treg differentiation (Figures 1D,E), with no obvious effect on cell proliferation (Figure 1F). Thus, by using two structurally different small molecule inhibitors, we affirmed that inhibition of Cdk8/Cdk19 could promote TGF-β-induced Treg cell differentiation.

### Cdk8/Cdk19 Blockade Upregulates the Expression of Treg Signature Genes and Enhances the Suppressive Function of Treg Cells *in vitro*

**Figure 2:**
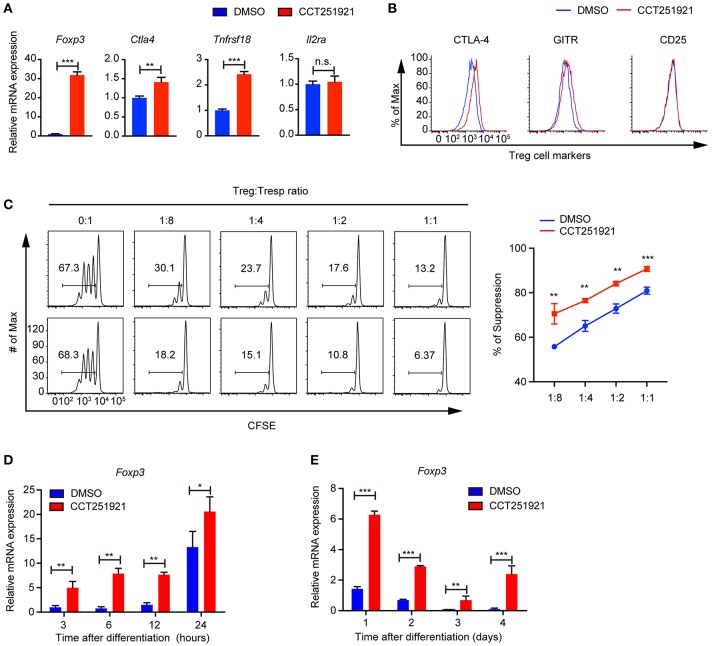
Blockade of Cdk8/Cdk19 promotes the expression of Treg signature genes and enhances the Treg suppressive function *in vitro*. **(A)** The relative mRNA expression level of *Foxp3, Ctla4, Tnfrsf18* (GITR), and *IL2Ra* compared to β*-actin* from CD4 T cells after 3 days *in vitro* iTreg differentiation treated with DMSO or CCT251921 (0.1 μM). **(B)** The expression of CTLA-4, GITR, and CD25 in CD4^+^Foxp3^+^ Treg cells as shown in **(A)**. The FACS plots are representative for three independent experiments. **(C)** The *in vitro* suppressive function of Treg cells from DMSO or CCT251921 treated groups analyzed by Tresp (responder T) cell proliferation (left) and statistical summary of suppression efficiency (right). (*N* = 3 mice; representative results are shown; means ± SD, **p < 0.01, ***p < 0.001, per two-sided *t*-test). **(D)** The mRNA expression level of *Foxp3* compared to β-actin from CD4 T cells at indicated hours after *in vitro* iTreg differentiation treated with DMSO or CCT251921 (0.1 μM). **(E)** The mRNA expression level of *Foxp3* compared to β-actin from CD4 T cells at indicated days after *in vitro* iTreg differentiation treated with DMSO or CCT251921 (0.1 μM). All the realtime results are summary of 3 mice per group from three independent experiments, Mean ± SD, *p < 0.05, **p < 0.01, ***p < 0.001, *n.s*. not significant, by two-sided *t*-test.

Next, we examined the Treg signature gene expression after inhibiting Cdk8/Cdk19 activity by realtime PCR. Consistent with the increased Foxp3^+^ Treg population by FACS analysis, we found that CCT251921 treatment drastically increased the mRNA level of Foxp3 (Figure 2A). In addition to the master regulator Foxp3, Treg cells also express a number of signature genes, such as *Il2ra* (encoding CD25), *Clta4* (encoding CTLA-4), and *Tnfrsf18* (encoding GITR). The expression of these signature genes is critical for the proper suppressive function of Treg cells (1). As all the T cells upregulate CD25 expression after activation in cell culture, we did not see any difference in CD25 expression between CCT251921 and DMSO group. However, the mRNA expression of other Treg signature genes such as CTLA-4 and GITR were significantly increased in CCT251921 group when compared with DMSO group (Figure 2A). The increased protein expression of CTLA-4 and GITR were further affirmed by FACS analysis (Figure 2B), indicating Cdk8/Cdk19 inhibition indeed enhanced the Treg signature gene expression. To validate whether the increased Treg signature expression could also enhance the suppressive function of Treg cells after Cdk8/Cdk19 inhibition, we performed *in vitro* Treg suppression assay. We found the proliferation of responsive CD4 T cells were more significantly inhibited by CCT251921-treated Treg cells as compared to DMSO-treated Treg cells (Figure 2C).

As Foxp3 is the master regulator of Treg cells and the upregulation of Foxp3 is the most drastic phenomenon after Cdk8/Cdk19 inhibition, we investigated time course of Foxp3 induction during Treg cell differentiation using sorted Naïve T cells and found CCT251921 treatment dramatically enhanced Foxp3 mRNA expression as early as 3 h after TCR activation (Figure 2D) and maintains high during the following days of Treg differentiation (Figure 2E). Those results suggested that enhanced Treg differentiation by Cdk8/Cdk19 inhibition is through the transcriptional regulation of Foxp3.

### Cdk8/Cdk19 Inhibition Sensitizes TGF-β Signaling and Promotes Foxp3 Expression

**Figure 3:**
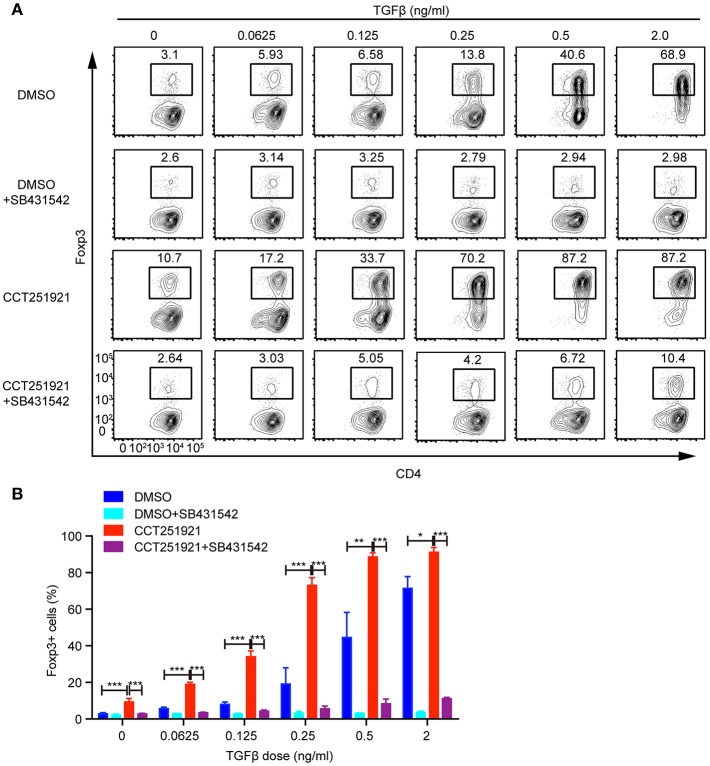
Blocking Cdk8/Cdk19 activity sensitizes TGF-β signaling to promote Treg differentiation. **(A)** Purified CD4^+^ T cells were cultured under Treg cell polarizing condition with different dose of TGF-β in the presence of DMSO, or CCT251921 (0.1 μM), with/out SB431542 (1 μM). Treg population was assessed by Foxp3 intracellular staining and flow cytometry. The FACS plots are representative for three independent experiments. **(B)** Statistical analysis of Treg population in **(A)**. Mean ± SD, *p < 0.05, **p < 0.01, ***p < 0.001, by two-sided *t*-test.

TGF-β signaling plays an instructional role in the differentiation of induced Treg cells (15). We asked whether blockade of Cdk8/Cdk19 might enhance the sensitivity of TGF-β signaling in T cells and thus promote Treg differentiation. To test this hypothesis, we used different doses of TGF-β during Treg differentiation in the presence of DMSO or CCT251921. The percentage of Foxp3^+^ Treg cells increased gradually in a TGF-β dose-dependent manner in DMSO group, while CCT251921 treatment drastically augmented Treg cell population even at very low dose of TGF-β (Figures 3A,B). To further confirm whether TGF-β signaling is indispensable for Cdk8/Cdk19 inhibition promoted Treg cell differentiation, a TGF-β receptor I inhibitor SB431542 was added into the *ex vivo* Treg differentiation system containing DMSO or CCT251921. We found that blockade of TGF-β signaling prevented Treg differentiation regardless CCT251921 treatment (Figures 3A,B). Those results indicated that enhanced Treg cell differentiation by Cdk8/Cdk19 blocking was dependent on TGF-β signaling and inhibition of Cdk8/Cdk19 increased the sensitivity of T cells in response to TGF-β and thus Treg differentiation.

### Cdk8/Cdk19 Inhibition Sensitizes TGF-β Signaling via Attenuating IFN-γ Signaling and Enhancing Smad2/3 Phosphorylation

**Figure 4:**
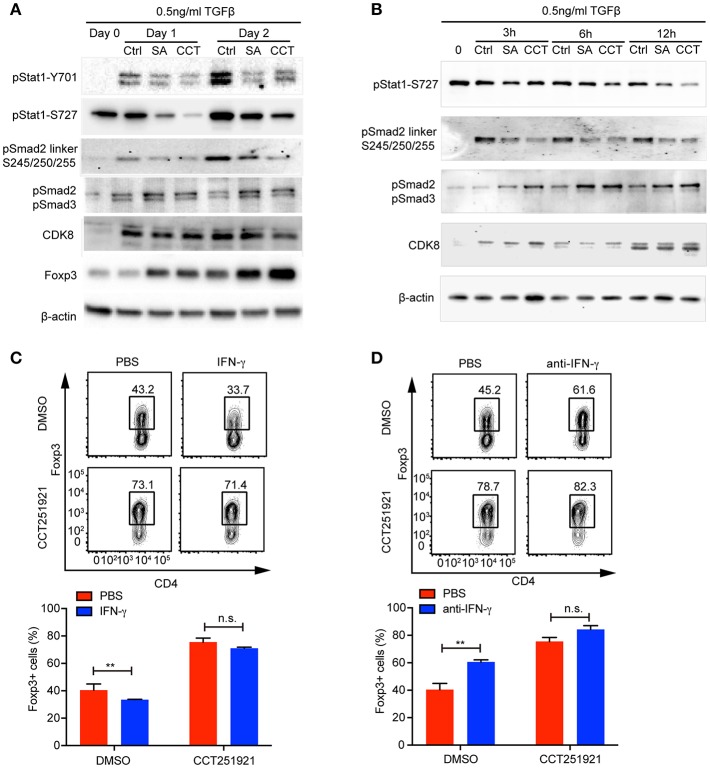
Cdk8/Cdk19 inhibition promotes Treg differentiation by suppressing IFN-γ signaling and activating TGF-β-Smad signaling. **(A)** The phosphorylation of Stat1 (pStat1-Y701, pStat1-S727), Smad2/3 (pSmad2, pSmad3) and Smad2 linker (pSmad2 Ser245/250/255), and protein expression of CDK8 and Foxp3 were analyzed by western blot at day 1 and day 2 of iTreg differentiation in the presence of DMSO, Senexin A (0.5 μM, SA), or CCT251921 (0.1 μM, CCT). The image was representative for at least three independent experiments. **(B)** The phosphorylation of Stat1 (pStat1-S727), Smad2/3 (pSmad2, pSmad3) and Smad2 linker (pSmad2 Ser245/250/255), and protein expression of CDK8 at early time points (0, 3, 6, and 12 h) of iTreg differentiation in the presence of DMSO, Senexin A (0.5 μM, SA), or CCT251921 (0.1 μM, CCT). The image was representative for at least three independent experiments. **(C)** Purified CD4^+^ T cells were cultured under iTreg cell polarizing condition (1 ng/ml of TGF-β and 20 U/ml of IL-2) in the presence of DMSO, or CCT251921 (0.1 μM), with/out IFN-γ (20 ng/ml). Treg population was assessed by Foxp3 intracellular staining and flow cytometry. **(D)** Purified CD4^+^ T cells were cultured under iTreg cell polarizing condition (1 ng/ml of TGF-β and 20 U/ml of IL-2) in the presence of DMSO, or CCT251921 (0.1 μM), with/out anti-IFN-γ (5 μg/ml). Treg population was assessed by Foxp3 intracellular staining and flow cytometry. For **(C,D)**, the FACS plots are representative for more than three independent experiments. Mean ± SD, *n.s*. not significant, **p < 0.01, by two-sided *t*-test.

While inflammatory cytokines could inhibit TGF-β signaling and Treg differentiation (16), the blockade of IFN-γ signaling enhances Treg cell differentiation in a Stat1 dependent way (17). In addition, Cdk8 has been reported to modulate IFN-γ induced Stat1 phosphorylation (7). Hence, we hypothesized that inhibition of Cdk8/Cdk19 might suppress IFN-γ signaling and relieve its antagonistic effect on TGF-β signaling. While the expression of IFN-γ was induced during Treg cell differentiation compared to unstimulated naïve CD4 T cells, Cdk8/Cdk19 inhibition only partially reduced the IFN-γ expression in the presence of high dose of TGF-β (Supplementary Figure 1A), indicating downstream of IFN-γ might be affected. Indeed, we observed a drastic decrease of Stat1 phosphorylation (both Y701 and S727) in the presence of CCT251921 or Senexin A at day 1 and day 2 of Treg cell differentiation (Figure 4A), indicating Cdk8/Cdk19 inhibition blocked IFN-γ induced Stat1 phosphorylation in T cells. To our surprise, Stat1-S727 was constitutively phosphorylated in the resting CD4 Naïve T cells which might be related to its nuclear translocation and T helper cell differentiation reported by Maldonado et al. (18). Moreover, the expression of Foxp3 was also upregulated simultaneously, which suggested that suppression of IFN-γ signaling by Cdk8/Cdk19 blockade could release its inhibition on TGF-β induced Foxp3 expression. Since Foxp3 mRNA expression was found upregulated as early as 3 h with Cdk8/19 blockade, we wonder whether IFN-γ signaling was affected at the very early stage of Treg cell differentiation. Western blot results showed that phosphorylation of Stat1 at Ser727 was significantly decreased after Senexin A or CCT251921 treatment from 3 to 12 h (Figure 4B). We found Cdk8 protein expression was low in naïve CD4 T cells and started to increase at 3 h after T cell activation and peaked at 24 h (Figures 4A,B). Additionally, Cdk8 protein expression was not affected by inhibitors treatment during Treg cell differentiation (Figures 4A,B).

To further address whether enhanced Foxp3 expression by Cdk8/Cdk19 blockade was associated to attenuated IFN-γ signaling, we added IFN-γ into the differentiation system and found that IFN-γ treatment suppressed Treg cell differentiation in control group, while treatment with CCT251921 significantly increased Treg cell differentiation and addition of IFN-γ had little effect on Foxp3^+^ Treg percentage enhanced by Cdk8/Cdk19 blockade (Figure 4C). Furthermore, blockade of IFN-γ signaling by IFN-γ neutralizing antibody enhanced Treg differentiation in control group but not CCT251921 treatment group (Figure 4D), which indicated that Cdk8/Cdk19 inhibition sensitized TGF-β signaling at least partially dependent on directly preventing IFN-γ induced Stat1 phosphorylation.

The phosphorylation of the tail region of regulatory Smad2/3 induced by TGF-β signaling is essential for its transcriptional activity and hence Foxp3 expression. On the other hand, the phosphorylation of linker domain promotes Smad ubiquitination and degradation, which has been reported to be regulated by Cdk8 in tumor cell lines (8). To analyze whether inhibition of Cdk8/Cdk19 could directly block Smad2 linker phosphorylation in T cells, we found TCR activation induced Smad2 linker phosphorylation at Ser245/250/255 and both Senexin A and CCT251921 prevented the linker phosphorylation (Figures 4A,B), which significantly increased its stability (Supplementary Figure 1B). Furthermore, the phosphorylation of Smad2/3 tail region was upregulated as early as 3 h during Treg cell differentiation upon Senexin A or CCT251921 treatment as compared to DMSO treatment (Figures 4A,B). Taken together, our result showed that the attenuation of IFN-γ signaling and upregulation of Smad2/3 activation contributed to enhanced Treg differentiation by Cdk8/Cdk19 inhibition.

### Treatment With Cdk8/Cdk19 Inhibitor CCT251921 Ameliorates Symptoms of EAE

**Figure 5:**
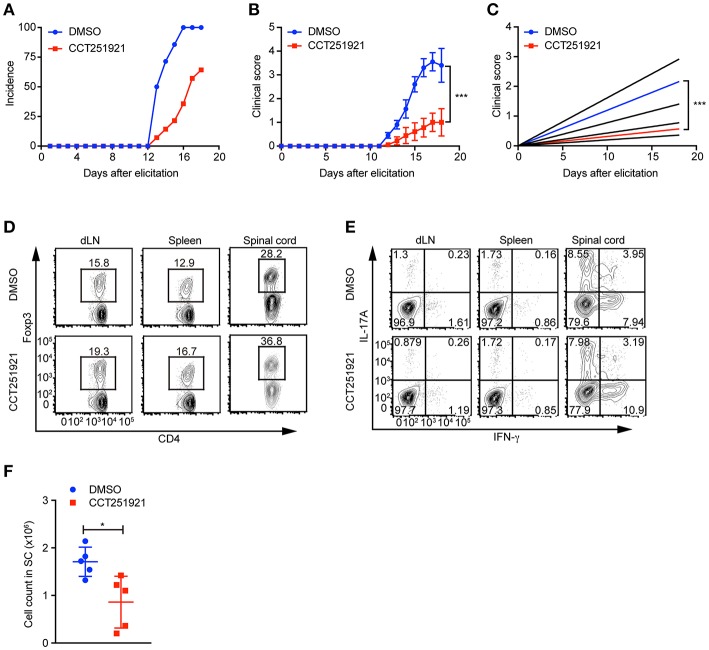
Treatment with Cdk8/Cdk19 inhibitor ameliorates EAE symptoms. **(A–C)** The disease incidence **(A)** and the recorded clinical scores **(B)** and the linear-regression analysis **(C)** of mice with indicated treatments at different time points after EAE elicitation. *N* = 10 mice for DMSO group; *N* = 9 mice for CCT251921 group. Mean ± SEM, ***p < 0.001, by two-way multiple-range ANOVA. **(D,E)** The population of CD4^+^Foxp3^+^ Treg cells **(D)** and IFN-γ, IL-17A-producing CD4^+^ T cells **(E)** in draining lymph nodes (dLN, left), spleen (middle) and spinal cord (right) at day 18 of EAE treated either with DMSO or CCT251921. **(F)** Total lymphocyte number recovered in the spinal cord of EAE mice with indicated treatment. *N* = 5 mice, Mean ± SD, *p < 0.05, by two-sided student's *t*-test.

Treg cells negatively regulate immune responses and maintain immune tolerance (1). It has been reported that Treg cells are critical to control central nervous inflammation in EAE (19). To investigate whether Cdk8/Cdk19 inhibition promoted Treg cells were functionally competent to suppress autoimmunity *in vivo*, we analyzed the inhibitory effect of CCT251921 in EAE model. Firstly, mice were immunized with MOG peptide and treated with pertussis toxin (PTX) to establish EAE model. Secondly, mice were divided into two groups randomly and administrated with CCT251921 in PBS by daily *i.p*. injection (1 mg/Kg body weight) or same amount of diluted DMSO in control group, respectively. We found most of the mice in control group rapidly developed very severe EAE symptoms from 12 days after immunization, whereas mice treated with CCT251921 showed less incidence of EAE (Figure 5A). Importantly, the average clinical score and regression clinical score were also much lower in CCT251921 group than those in DMSO group (Figures 5B,C). These results demonstrated that the inhibition of Cdk8/Cdk19 by CCT251921 ameliorates autoimmunity in EAE model.

To further examine the impact of inhibitor treatment on Treg cells, we analyzed the Treg cell population in draining lymph nodes, spleen, and spinal cord of mice at day 18 of EAE. We found there was a significant increase of Foxp3^+^ Treg population both in periphery lymphoid tissues and spinal cord in CCT251921 group as compared to DMSO group (Figure 5D), while there was no significant change in the population of IFN-γ and IL-17A producing CD4 T cells (Figure 5E). We also found the total number of infiltrating lymphocytes from spinal cord in CCT251921 treated mice was significantly less than DMSO group (Figure 5F), indicating blockade of Cdk8/Cdk19 is sufficient to promote Treg differentiation and suppress autoimmune symptoms in EAE.

## Discussion

As the major regulator of adaptive immunity, manipulating Treg cells has emerged as an attractive therapeutic strategy for various diseases both in autoimmunity and tumor immunology (2). Here we demonstrated that pharmacological inhibition of Cdk8/Cdk19 promoted the differentiation of Treg cells and the expression of Treg signature genes. Further analysis indicated that blockade of Cdk8/Cdk19 sensitized TGF-β signaling to promote the transcription of Foxp3 gene, which was partially via inhibiting IFN-γ signaling and activating Smad2/3. Importantly, our animal study revealed that treatment with Cdk8/Cdk19 inhibitor CCT251921 increased Treg population and suppressed autoimmune symptoms in MOG peptide-induced EAE model. The identification of Cdk8/Cdk19 in regulating Treg differentiation and immune suppression will broaden our understanding of Cdk8/Cdk19 in T cell regulation and provide a valuable target for reprogramming Treg cells.

Dual inhibition of Cdk8/Cdk19 by small molecule inhibitor has been reported to up-regulate IL-10 production in dendritic cells and macrophages (13), indicating Cdk8/Cdk19 are involved in innate immunity regulation. By using two structurally different small molecule inhibitors, we demonstrate that inhibition of Cdk8/Cdk19 promotes Treg differentiation and augments immune suppression. Consistent with our expectations, the differentiation of Th1 was also slightly decreased in a dose-dependent manner since interference of IFN-γ signaling by Cdk8/Cdk19 inhibition (data not shown). More interestingly, in another TGF-β-dependent differentiation model, we found that treatment with Cdk8/Cdk19 inhibitor promoted Th17 differentiation with low dose TGF-β while inhibited Th17 differentiation and proliferation under condition of high dose TGF-β (data not shown). Thus, from Th17 differentiation model, those results indirectly reflected that blocking Cdk8/Cdk19 activity sensitizes and amplifies TGF-β signaling indeed. Our study for the first time demonstrates the regulating role of Cdk8/Cdk19 in T cell differentiation, especially Treg cell differentiation via sensitizing TGF-β signaling.

Human Cdk8 and its paralog Cdk19 share 77% identical (82% similar) amino acids with 97% identity in the kinase domain. Cdk8 was firstly identified as oncogene in colon cancer by regulating β-catenin signaling (6). Later studies indicate that Cdk8 can phosphorylate a number of substrates that regulates different signaling pathways, such as Stat1 in response to IFN-γ (7) and linker region of Smad proteins in BMP signaling and TGF-β signaling (8). Compared with Cdk8, there is little known about the function of Cdk19. The next important question is to identify the specific role of Cdk8 or Cdk19 in regulating Treg differentiation either by genetic knockout mice or CRISPR-Cas9 knockout system. Since both environmental and cell intrinsic stimuli could influence and modify TGF-β signaling, our results show that Cdk8/Cdk19 regulates TGF-β signaling and Treg differentiation partially by inhibiting IFN-γ-Stat1 pathway and promoting Smad2/3 activation. However, we also observed the IFN-γ-Stat1 signaling independent enhancement of Treg differentiation. Comprehensively investigating the mechanisms of Cdk8/Cdk19 inhibition on Treg differentiation will further advance our knowledge on the regulation of Treg cell differentiation.

Cdk8 was initially identified as oncogene in colon cancer (6). Later on Cdk8 was reported to play a role in many other cancers such as pancreatic cancer (20), breast cancer (10, 21), and melanoma (22). The study on Cdk8 has been mainly focused in the field of tumor biology. And a number of inhibitors targeting Cdk8 have been developed to treat cancers (23). Currently, a Cdk8 inhibitor, BCD-115, had been used in clinical trials to treat advanced and metastatic breast cancer (NCT03065010). In the present study, we demonstrated that Cdk8 inhibition promoted Treg cell differentiation and suppressed autoimmunity in the EAE model. Therefore, combining with Treg inhibition might improve the therapeutic effect of Cdk8/Cdk19 inhibitors in cancer therapy. In addition, investigating the role of Cdk8/Cdk19 inhibition on other autoimmune diseases such as graft-vs.-host disease, SLE, and rheumatoid arthritis (RA) will further confirm the function of Cdk8/Cdk19 in adaptive immunity regulation and diversify its application in the field of autoimmunity.

## Materials and Methods

### Mice

WT (C57BL/6) and CD45.1 congenic wild type mice were on the C57BL/6 genetic background. The mice were housed and bred under specific pathogen-free condition in the animal facility at the University of North Carolina at Chapel Hill or Xuzhou Medical University. All animal experiments were approved by Institution Animal Care and Use Committee of the University of North Carolina at Chapel Hill and the Animal Care and Use Committee of Xuzhou Medical University.

### Lymphocyte Isolation, Treg Differentiation and Proliferation

Lymphocytes were isolated from peripheral lymph nodes and spleen of age- and sex- matched mice and purified with CD4 microbeads (L3T4, Miltenyi Biotec). Purified CD4^+^ T cells were cultured in RPMI 1640 medium containing 10% FBS, 1% penicillin-streptomycin and 75 μM of β-Mercaptoethanol and activated with plate-coated 2.5 μg/ml CD3 (145-2c11, Bio-X-Cell) and 1 μg/ml CD28 (37.51, Bio-X-Cell) antibodies. For Treg differentiation, designated doses of TGF-β (0.01–2 ng/ml) and IL-2 (20 U/ml) were added into culture medium. CCT251921 (HY-19984, MedChem Express) and Senexin A (487510, Tocris Bioscience) were used to block Cdk8/Cdk19 activity. TGF-β receptor I (Alk5) inhibitor SB431542 (S1067, Selleckchem) was used to block TGF-β signaling. For proliferation assay, 2 μM of CFSE (C1157, Life Technologies) was used to label CD4^+^ T cells before differentiation.

### Flow Cytometry and Cell Sorting

Fluorescence-conjugated antibodies for CD4 (RM4-5), IFN-γ, IL-17A (TC11-18H10.1), CD45RB (MEM-55), and CD90.1 (H1.2F3) were purchased from Biolegend and CD25 (PC61.5) and FoxP3 (FJK-16s) were from eBioscience. For FACs staining, 0.5 – 1 × 10^6^ cells were collected and surface stained followed by intracellular staining after fixation and permeabilization according the manufacture's instruction (BD Bioscience). For intracellular cytokine staining, lymphocytes were stimulated for 4 h with 50 ng/mL of PMA (phorbol 12-myristate 13-acetate) and 1 mM ionomycin in the presence of brefeldin A. Stained cells were analyzed on the Canto II ™ (BD biosciences) and FACS data were analyzed with FlowJo software (TreeStar). For naïve T cell sorting, CD4^+^ T cells were enriched by MACs and then stained with fluorescence-conjugated antibodies and CD4^+^CD25^−^CD45RB^hi^ naïve cells were sorted on the Moflow cell sorter (Dako cytomation, Beckman coulter) by the flow facility of University of North Carolina at Chapel Hill or the BD FACSAria™ III sorter in Xuzhou Medical University.

### *In vitro* Treg Suppression Assay

To assess the efficacy of Treg cell-mediated immune suppression *in vitro*, 1 × 10^5^ of sorted naïve CD4 responder T (Tresp) cells from CD45.1 congenic WT mice were labeled with CFSE and mixed with varying amounts (as indicated) of Treg suppressor cells. Cell mixtures were stimulated with soluble anti-CD3 antibody (0.04 μg/ml) in the presence of 4 × 10^5^ irradiated (3,000 cGy) T cell-depleted splenocytes. The proliferation of Tresp cells was assessed by CFSE dilution detected by flow-cytometry 72 h after activation. The suppression efficiency was calculated by the formula: [1—(Tresp proliferation with Treg)/(Tresp proliferation without Treg)] × 100.

### RNA Preparation and Real-Time PCR

Total RNA was isolated from T cells by TRIzol reagent (Invitrogen) following manufacturer's instruction, and then reverse-transcribed into cDNA with iScript™ cDNA Synthesis Kit (BioRad). Quantitative real-time PCR was performed on ABI9700 PCR system with Taqman-probe sets purchased from Applied Biosystems and Integrated DNA Technologies (IDT). The relative mRNA expression of indicated genes was calculated based on the expression of β*-actin*.

### Western Blot

Cells were lysed in RIPA lysis buffer containing protease inhibitor cocktail and phosSTOP (Roche). Cell lysate was treated with 2 × Laemmli sample buffer (Bio-Rad) and denatured at 100°C for 10 min. Protein extracts were separated by 4–15% SDS-PAGE gel (Bio-Rad) and transferred to the PVDF membrane (Millipore) and analyzed by immuno-blotting with following antibodies: β-actin (I-19, Santa Cruz), CDK8 (P455, CST), pStat1-Y701 (58D6, CST), pStat1-S727 (D3B7, CST), pSmad2 linker (Ser245/250/255, CST), pSmad2 (Ser465/467)/Smad3 (Ser425/427) (D27F4, CST), and FoxP3 (eBio7979, eBioscience). Images of western blot were taken by Bio-Rad imaging system and had been cropped according to the molecular weight for presentation.

### Experimental Autoimmune Encephalomyelitis (EAE)

To elicit EAE model, age (8–12 weeks old) and gender matched mice were subcutaneously immunized (s.c.) with 50 μg of MOG35-55 peptide (MEVGWYRSPFSR VVHLYRNGK, AnaSpec) and 500 μg of M. tuberculosis (Difco) emulsified in IFA (Difco). In addition, each mouse was received 200 ng of Pertussis Toxin (List Biological Laboratories) intraperitoneally (*i.p*.) on day 0 and day 2, respectively. Then, 1 mg/kg CCT251921 or same of volume DMSO in PBS were *i.p*. injected for treatment. The severity of EAE was monitored and graded on a clinical score of 0 to 5 by the following standard 0 = No clinical signs, 1 = Limp tail, 2 = Para-paresis (weakness, incomplete paralysis of one or two hind limbs), 3 = Paraplegia (complete paralysis of two hind limbs), 4 = Paraplegia with forelimb weakness or paralysis, 5 = Moribund or death. The statistical significance was analyzed by two-way multiple-range ANOVA test.

After EAE elicitation, mice were sacrificed and perfused with ice-cold phosphate buffered saline containing 20 U/ml of heparin. Spinal cord was separated from spine columns after removal of all tissues. The isolated spinal cords were minced and digested with 1 mg/ml collagenase D (Sigma) for 45 min at 37°C. The digested tissues were filtered with 40 μm strainer and centrifuged, thereafter resuspended into 38% percoll (Sigma) and centrifuged at 2,000 rpm for 20 min to separate lymphocytes. Lymphocytes were then isolated and subjected to FACs analysis.

### Statistical Analysis

Data analysis was processed by Prism (GraphPad, San Diego) and presented as Mean ± SD unless otherwise described. All experiments were repeated at least 3 times and representative figures were shown in the article. Statistical significance was determined by Student's *t*-test and two-way multiple-range ANOVA test. A *p*-value of <0.05 was considered statistically significant.

## Data Availability

The datasets generated for this study are available on request to the corresponding author.

## Author Contributions

ZG and GW designed and performed the experiments. YL performed the experiments. ZG, GW, YW, and JZ analyzed the data and wrote the manuscript.

### Conflict of Interest Statement

The authors declare that the research was conducted in the absence of any commercial or financial relationships that could be construed as a potential conflict of interest.
