# Supplementary figures and images for: Inhibition of Cdk8/Cdk19 Activity Promotes Treg Cell Differentiation and Suppresses Autoimmune Diseases
Source: Front Immunol. 2019 Aug 20;10:1988. doi: 10.3389/fimmu.2019.01988 (PMC6736578; doi:10.3389/fimmu.2019.01988)

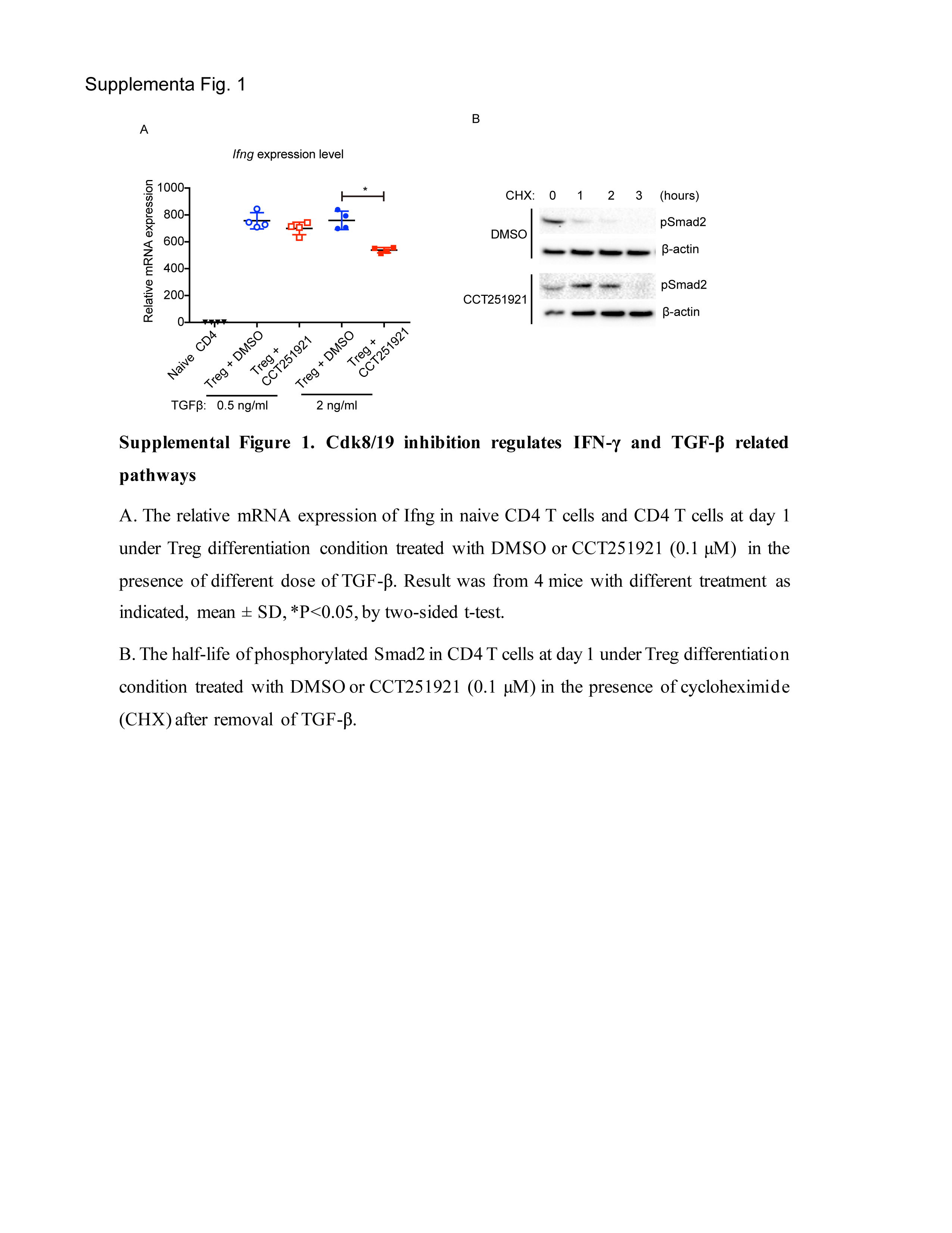

Supplement: Supplementary file 1 [file Image_1.TIF]
